# Non-Typhoidal *Salmonella* at the Human-Food-of-Animal-Origin Interface in Australia

**DOI:** 10.3390/ani10071192

**Published:** 2020-07-14

**Authors:** Hamid Reza Sodagari, Penghao Wang, Ian Robertson, Ihab Habib, Shafi Sahibzada

**Affiliations:** 1 School of Veterinary Medicine, College of Science, Health, Education and Engineering, Murdoch University, Perth 6150, Australia; Hamidreza.Sodagari@murdoch.edu.au (H.R.S.); P.Wang@murdoch.edu.au (P.W.); I.Robertson@murdoch.edu.au (I.R.); 2 Veterinary Medicine Department, College of Food and Agriculture, United Arab Emirates University (UAEU), Al Ain P.O. Box 1555, UAE

**Keywords:** Australia, antimicrobial resistance, food safety, zoonoses, one health

## Abstract

**Simple Summary:**

The present review of the literature highlights the epidemiology of non-typhoidal *Salmonella* at the human–food-of-animal-origin interface, as well as the antimicrobial resistance trends of non-typhoidal *Salmonella* isolates in different Australian states and territories over the past two decades, improving our understanding of how to better control and prevent human salmonellosis outbreaks in Australia.

**Abstract:**

Non-typhoidal *Salmonella* is a major zoonotic pathogen that plays a significant role in foodborne human salmonellosis worldwide through the consumption of contaminated foods, particularly those of animal origin. Despite a considerable reduction in human salmonellosis outbreaks in developed countries, Australia is experiencing a continuous rise of such outbreaks in humans. This review of the literature highlights the reported non-typhoidal *Salmonella* outbreaks in humans as well as the occurrence of the pathogen in foods from animal sources throughout Australia. Non-typhoidal *Salmonella* infections from food animals are more often associated with at-risk people, such as immunocompromised and aged people or children. Although several animal-sourced foods were recognised as the catalysts for salmonellosis outbreaks in Australia, egg and egg-based products remained the most implicated foods in the reported outbreaks. This review further highlights the antimicrobial resistance trends of non-typhoidal *Salmonella* isolates at the human–food interface, with a focus on clinically important antimicrobials in humans, by collating evidence from previous investigations in Australia. The rise in antimicrobial-resistant *Salmonella*, especially to antimicrobials commonly prescribed to treat human salmonellosis, has become a significant global public health concern. However, the overall prevalence of antimicrobial resistance in Australia is considerably lower than in other parts of the world, particularly in terms of critically important antimicrobials for the treatment of human salmonellosis. The present review adds to our understanding of the global epidemiology of non-typhoidal *Salmonella* with emphasis on the past few decades in Australia.

## 1. Introduction

Non-typhoidal *Salmonella* is a Gram-negative, facultative anaerobic, rod-shaped and motile bacterium belonging to the *Enterobacteriaceae* family. The bacteria are chemo-organotrophic and grow optimally at 37 °C. Non-typhoidal *Salmonella* play a significant role in foodborne human salmonellosis worldwide [1] and can be transmitted to humans particularly through the consumption of foods of animal origin, including eggs and poultry meat, as well as through direct contact with animals or their environments [2,3].

More than 2500 serovars of *Salmonella enterica* have been identified, of which many can cause human infections. However, non-typhoidal serovars, especially Enteritidis and Typhimurium, are the most commonly isolated serotypes in human infections [4]. Salmonellosis in humans is commonly characterised by diarrhoea, abdominal cramps, fever and vomiting [5]. Although most non-typhoidal *Salmonella* infections are associated with self-limiting gastroenteritis, they have the potential to cause fatal infections among infants, young children, older adults and immunocompromised individuals [6]. The majority of non-typhoidal *Salmonella* serovars are pathogenic as a result of their ability to invade, replicate and survive in human host cells [7]. Several mechanisms that are not yet fully understood are associated with the pathogenesis of *Salmonella* infection. It has been shown that the *Salmonella* pathogenicity islands (SPI-1 to SPI-17) and gene clusters encode for the structures involved in the invasion process [8], and play a significant role in the development of the disease. Additionally, fimbrial operons (*bcf*, *csg*, *stb*, *sth*, *sti*) and colonisation factors (*misL*, *bapA*, *sinH*) have been attributed to the pathogenesis of *Salmonella* infection [9]. Australia has relatively high rates of notified human salmonellosis in comparison to several other industrialised countries [10]. Despite a considerable reduction in the incidence of human salmonellosis in developed countries, this rate continues to rise every year in Australia [11,12,13]. The number of reported cases of human salmonellosis in Australia (69.3 cases per 100,000) [14] was approximately four times higher than in the USA (15.45 cases per 100,000) [11]. On the other hand, although the number of Salmonellosis is reported to be high in Australia, antimicrobial resistance amongst *Salmonella* isolates is considered to be lower in comparison with other parts of the world.

Non-typhoidal *Salmonella* infections are generally understood to be acquired from animal reservoirs. Although salmonellosis outbreaks in Australia have been linked with a variety of foods of animal origin, such as red meat [14], poultry meat [15] and seafoods [16,17], egg and egg-based products are reportedly the most implicated foods in the majority of non-typhoidal human salmonellosis outbreaks (59%) in Australia [18,19,20].

Over the past 20 years, there have been increasing reports concerning the resistance of non-typhoidal *Salmonella* to a range of antimicrobials, including fluoroquinolones and extended spectrum cephalosporins, as the first-line antimicrobials for the treatment of severe human salmonellosis [21,22]. These reports have prompted both the World Health Organization and the Centers for Disease Prevention and Control to consider *Salmonella* resistant strains as a major public health threat [23,24].

This review focuses on the epidemiology of non-typhoidal *Salmonella* in humans, foods-of-animal-origin in Australia and antimicrobial resistance patterns. The review aims at clarifying (a) non-typhoidal human salmonellosis outbreaks across different Australian states and territories, (b) the prevalence of *Salmonella* in foods of animal origin and (c) *Salmonella*’s resistance to antimicrobial agents, with a focus on key antimicrobials, such as fluoroquinolones and cephalosporins, for treating human infection.

## 2. Non-Typhoidal *Salmonella* Outbreaks in Humans

In this review, we collate evidence from previous literature to facilitate data consolidation and the regional comparisons of different Australian states and territories (New South Wales [NSW], Victoria [VIC], South Australia [SA], Western Australia [WA], Tasmania [TAS], Queensland [QLD], the Australian Capital Territory [ACT] and the Northern Territory [NT]) over the past two decades. Table A1 provides a summarised report on most of the published data on human salmonellosis outbreaks linked to foods of animal origin in Australia.

It seems that among the different *Salmonella* serovars, *S.* Typhimurium was responsible for over 40% of outbreaks in all Australian states and territories. However, in some areas with tropical climatic zones, such as the NT, QLD and WA, non-Typhimurium *Salmonella* serovars were reported to be responsible for the majority of human salmonellosis outbreaks [25]. 

### 2.1. New South Wales

Several salmonellosis outbreaks have been reported in NSW. The range of salmonellosis cases vary between the outbreaks (Table A1), from ten cases in an aged-care facility in 2008 [26] to 365 bakery-linked cases in 2007 [27]. Egg-based products were the suspected sources of *S.* Typhimurium in both outbreaks. The bakery-linked outbreak in 2007 was one of the largest point source outbreaks of *Salmonella* in Australia. A similar outbreak with a higher number of cases (n = 774) occurred ten years earlier as a result of the consumption of Vietnamese pork rolls sourced from a local hot bread shop [28]. Another large point source, a bakery-linked *S.* Typhimurium outbreak associated with the consumption of Vietnamese-style pork, chicken and salad rolls, was reported in Sydney in 2011 [29]. In this outbreak, 77% and 24% of 83 detected cases, respectively, sought medical attention and were hospitalised. The researchers proposed that extensive cross-contamination throughout the bakery, arising from a poor understanding of food-handling practices, played an important role in these large outbreaks [29]. Four years later, in 2015, another bakery-linked outbreak connected to Vietnamese bread rolls (containing pork or chicken with chicken-liver pâté and raw egg mayonnaise) was identified in southwestern Sydney [30]. However, the number of cases (n = 26) in this outbreak was much smaller than that of the two previous similar outbreaks.

In NSW, *S.* Typhimurium outbreaks relating to food at sports activities were also identified: an outbreak among 35 attendees of a high-profile sports club was associated with a lamb meal supplied by an external caterer in 2014 [31] and another, larger outbreak occurred at a privately catered barbeque at a sports club in 2009 [32], where 71 cases had consumed the raw egg mayonnaise used in a Russian salad. This larger outbreak indicated a high burden of illness: 76% and 18% of the identified cases required medical attention and hospitalisation, respectively [32]. The authors noted that, since sport teams might be at high risk of foodborne illness arising from mass catering at training, events or functions, strict food-safety principles are needed, particularly for large-scale events [31]. In February 2008, a point source outbreak of *S.* Typhimurium was identified with 44 cases aged three to 91 in the Central Coast region of NSW; eggs and egg-containing dishes sourced from a farm in NSW were the known sources of this outbreak [33]. The authors recommended the control of *Salmonella* at the farm level as a vital measure to reduce the burden of egg-related human salmonellosis in Australia.

In 2002, an outbreak caused by *S.* Potsdam was reported in 17 individuals after the consumption of shell egg-based salad dressings in a restaurant in NSW; two persons were hospitalised [34]. This serovar is occasionally reported in Australia, with a range of 40 to 60 cases identified annually since 1991. It was detected in egg samples from VIC in 1982 as well as in WA in 1985 and 1990 [35]. The 2002 outbreak highlighted the need for the daily preparation and storage of raw shell egg-based dressing and dishes under proper conditions at ≤ 5 °C, instead of at room temperature [34]. In another report in 2005, lamb’s liver was responsible for an *S.* Typhimurium outbreak, as shown in Table A1 [36]. Since the majority of *S.* Typhimurium outbreaks in Australia are associated with the consumption of egg and egg-based foods [19], the identification of lamb’s liver as the suspected source of the outbreak was uncommon. Little is known about the association between salmonellosis and lamb’s liver. There are only a few studies that have reported an association between offal and salmonellosis due to cross-contamination while handling cooked and raw meat products together [37,38].

A seafood-related *Salmonella* outbreak was also identified in Western Sydney in 2015 [16]. Six *S.* Agona outbreak cases were linked to the consumption of cooked-tuna sushi rolls purchased from a shopping complex. Not only is *S.* Agona recognised as a common cause of salmonellosis in both animals and humans globally [4] that is frequently traced to isolates from farmed livestock, vegetables [39,40,41] and factory-prepared foods [42,43,44,45], it was also among the top 15 most frequent *Salmonella* serovars in Australia during 2006 [4,46].

In June 2010, the Central Coast of NSW experienced an *S.* Typhimurium outbreak linked to a kebab takeaway shop, as described in Table A1 [47]. Chicken kebab rolls containing hummus and tabbouleh were identified as the outbreak sources. Since the hummus and tabbouleh were found to be positive for *S.* Typhimurium, the most likely explanation for the outbreak was cross-contamination. The authors [47] suggested improving food-handling practices to reduce the risk of contamination from poultry meat. 

*S.* Typhimurium is the predominant serovar in egg-borne salmonellosis outbreaks in Australia, which is remarkably different from other parts of the world, such as USA and Europe, where *S.* Enteritidis is a major cause of egg-related foodborne outbreaks [48]. Recently, more than 220 cases of illness reported in Australia (193 in NSW) were linked to *S.* Enteritidis outbreak in 2018–2019 [49]. Subsequently, *S.* Enteritidis was identified in 13 NSW poultry egg facilities and one Victorian poultry egg facility. All the properties confirmed to have had *S.* Enteritidis present were interconnected, in that people, eggs or equipment were moving between them [49].

### 2.2. Victoria

In 2001, lamb’s fry served in a hotel buffet was hypothesised to be the most plausible source of an *S.* Typhimurium outbreak in a rural region of VIC [14]. Among 18 suspected cases, two were admitted to hospital. In another report, a total of ten confirmed cases of *S.* Typhimurium were identified as linked to a bakery in Northern VIC in 2002 [50]. It seems likely that contaminated eggs or cream were the source of *Salmonella* in this outbreak. However, the origin and the mechanism of the contamination remained unrecognised. The researchers proposed that applying specific control measures particularly during pulping eggs and whipping cream such as hand washing and using covers to reduce splashing could significantly reduce the risk of cross-contamination during food preparation [50].

### 2.3. South Australia

From March 2017 to July 2018, a protracted outbreak of 25 cases of *S.* Hessarek infection associated with one brand of eggs was detected in SA; ten cases were admitted to hospital [51]. *S.* Hessarek has rarely been reported in Australia [51]. For the five-year period between 2012 and 2016, the rate of *S.* Hessarek cases in South Australians (3.1 per 100,000 persons) was more than seven times higher than the average national rate for Australia (0.4 per 100,000 persons) [52]. Finding an uncommon *Salmonella* serovar in this study suggests the need for the constant monitoring of the epidemiology of egg-associated human salmonellosis outbreaks in Australia.

In 2001, there were two *S.* Typhimurium outbreaks related to desserts containing raw eggs in SA: an outbreak linked to a Christmas function associated with tiramisu [53] and another outbreak in an aged-care facility resulting from the consumption of glazed pie [54], as shown in Table A1. The authors cautioned about the great potential risk of *Salmonella* infection from raw egg and egg-based products for vulnerable groups, including the elderly and the immunocompromised [53]. In the same year, an outbreak of an uncommon *Salmonella* serovar Zanzibar was identified in rural SA in two young adults aged 26 and 31 [55]. Although the source for this outbreak was not identified, both cases reported that they had eaten a chicken-based pasta dish at the same Italian restaurant.

In 2003, cheesecake containing eggs purchased from three different commercial food outlets was responsible for an outbreak of *S.* Typhimurium in six persons in SA [56]. In 2005, an outbreak of *S.* Typhimurium linked to catered luncheons was reported in Adelaide [57]. In this outbreak, with 32 laboratory-confirmed cases, a cross-contamination from the chicken to other ingredients commonly used in bread rolls was reported as the transmission route. Another *S.* Typhimurium outbreak in SA was reported among the attendees at a wedding reception in 2009, as shown in Table A1 [58]. In this outbreak, garlic aioli containing raw egg yolk was identified as the most plausible source of the contamination. The authors urged the need for more stringent regulations on the production and sale of eggs as well as product traceability.

### 2.4. Queensland

In 2002 in south-west Brisbane, an outbreak of *S.* Typhimurium was reported in a child-care centre [59]. Egg sandwiches were identified as the outbreak’s source, which affected 16 children under five years old. Since children under five are considered as an at-risk group, it was strongly recommended that the authorities ensure the supply of crack-free, clean eggs to all child-care centres. A cluster of *S.* Typhimurium associated with a restaurant was also reported in QLD in 2003. A significant association between illness and the consumption of roast pork was reported, indicating the pork as the most likely source of this outbreak [60].

In another outbreak in 2004, *S.* Singapore was responsible for illness in 11 young adults aged between 20 and 39 related to sushi consumption in Brisbane CBD [61]. Traditionally, sushi is not refrigerated and is often displayed and eaten at room temperature, which provides optimal conditions for the pathogen to grow. Nevertheless, sushi-related *Salmonella* outbreaks have rarely been described in the literature.

### 2.5. Tasmania

In 2005, a total of five outbreaks, including 125 laboratory-confirmed cases of *S.* Typhimurium infections, occurred as a result of the consumption of products containing raw egg related to group functions and restaurants in TAS [62]. These formed one of the largest egg-associated outbreaks in Australia. In this state, it has been reported that *S.* Typhimurium contributes 22% of the total *Salmonella* notifications and is the second most commonly reported *Salmonella* serovar after *S.* Mississippi [62]. Although 91% of cases in the five outbreaks were reported to be linked to food businesses supplied by a single egg farm, no eggs tested positive in the human food chain. According to another report in 2007, *S.* Typhimurium was identified in 18 individuals who had consumed bakery products, as well as in two persons following the consumption of egg-containing dishes at retail businesses [63]. The same egg supplier was recognised for both the bakery and the retail businesses.

In 2008, chicken sandwiches containing aioli were reported to be the suspected source for a total of 47 microbiologically confirmed *Salmonella* infections originating from a restaurant in southern TAS [63]. A number of food-safety issues were associated with both outbreaks, including improper storage temperate and poor hygienic measures such as lack of hand washing stations and paper towels. Moreover, a mixer used to blend aioli during the production process was found to have insufficient cleaning and sanitation. Epidemiological evidence noted the point source of infection in both outbreaks. The researchers observed the need for surveillance at all points in the food supply chain from production to consumption to remove food-safety risks.

### 2.6. Australian Capital Territory

In Canberra, eggs benedict served at a café was reported to be associated with *Salmonella* infection in 2012, as shown in Table A1 [64]. In addition, in Canberra in 2008, an *S.* Typhimurium outbreak was reported to be associated with the consumption of eggs and hollandaise sauce in a restaurant [65]. In 2009, Reynolds et al. [66] also reported a point source outbreak of *S.* Typhimurium linked to tiramisu containing raw egg with nine confirmed cases in Canberra. Contaminated eggs were the main catalyst for this outbreak, although the possibility of cross-contamination from another unknown source cannot be ignored. Egg-associated *salmonella* outbreaks in restaurants and cafés are not uncommon in ACT, and in likewise other states, and the majority are associated with cross contamination. The majority of these outbreaks can be reduced by sourcing eggs from trusted suppliers that perform routine microbiological testing and by improving communication between health departments and egg producers. 

### 2.7. Northern Territory

*S.* Litchfield is a common *Salmonella* serovar in northern Australia [67]. This serovar was also isolated from different non-human sources, including animals, food and the environment, in four different areas in Australia from 2002 to 2008 (personal communication, National Enteric Pathogens Surveillance Scheme, Microbiological Diagnostic Unit, Public Health Laboratory, University of Melbourne, 14 May 2010). According to a report by OzFoodNet, since 2001, two non-animal origins (papayas and cucumbers) have been associated with *S.* Litchfield outbreaks in Australia [67,68]. An outbreak of this serovar of animal origin was also identified in 76 individuals who ate barramundi fillets at a car rally in Darwin in 2009 [69].

Although a few *S.* Typhimurium outbreaks have been attributed to duck meat or eggs since 2001 (unpublished data from the OzFoodNet Outbreak Register; 2001–2015), there are no previously reported outbreaks linked to duck prosciutto in this country. As shown in Table A1, a *S.* Typhimurium outbreak occurred in 2015 following the consumption of duck prosciutto in a restaurant in Darwin [15]. Duck prosciutto was the most implicated food in this outbreak because of its high risk, relative to the other foods consumed by the affected cases. Nevertheless, no *Salmonella* was found in the tested samples of frozen duck fillet or frozen duck prosciutto, which is strong evidence for contamination during the drying and curing process.

Turtles are also reported as *Salmonella* reservoirs that may pose a public health risk of salmonellosis [70,71]; however, turtle meat is not commonly used in Australia, apart from in Top End Aboriginal communities [72]. An outbreak of *S.* Chester was reported following the consumption of a green turtle in a remote coastal Aboriginal community of the NT in 1998, with six hospitalisations among the 36 identified cases [73]. Another turtle-associated *Salmonella* outbreak was reported in the NT more recently in 2017 [17]. *S.* Muenchen was recognised as responsible for this outbreak in an Aboriginal community, which arose from the consumption of sea-turtle meat. The authors mentioned that the butchering and storing of meat at improper temperature, as well as the consuming of semi-cooked meat, might be responsible for such an outbreak.

## 3. Prevalence of Non-Typhoidal *Salmonella* in Foods of Animal Origin

Foods of animal origin, including meat, eggs, milk and other products, play a significant role in the daily diets of Australian people. In Australia, different rates of *Salmonella* occurrence have been reported as originating in foods of animal origin, from 0% in beef, sheep carcasses and eggs, to 54.5% in chicken meat, which is comparable to the reported rate for chicken meat samples in China (57%, 114/200) [74] and higher than the obtained result for Singapore (18.1%, 49/270) [75]. Differences in prevalence are also reported for other foods of animal origin in different parts of the world, such as retail beef in Malaysia (7.5%, 18/240) [76] and pork in the USA (3.3%, 7/209) [77]. Table A2 provides a summary of the *Salmonella* occurrence in foods of animal origin.

### 3.1. New South Wales

Chicken meat is the most consumed meat in Australia. NSW is the leading poultry-producing state, averaging 33% of national production in recent years [78]. In a baseline survey, *Salmonella* was detected in 47.7% of 549 chicken portions and carcasses sourced from retail markets [79]. Different *Salmonella* serovars detected in this investigation, of which *S.* Sofia was the most frequently identified, accounted for 35.3% of the isolates. Although *S.* Sofia is among the most prevalent serovar isolated from poultry, it is responsible for only 0.3% of reported human salmonellosis cases in Australia [79].

The samples collected during winter contained higher rates of *Salmonella* recovery (51.4%) than those sampled during summer (43.9%) [79]. Moreover, different recovery rates were reported based on product type and retail mode. *Salmonella* was present in 77.8%, 40% and 29.7% of whole birds collected from butcher shops, specialty shops and supermarkets, respectively [79]. The authors further identified no considerable differences between product type (skin-on and skin-off) and presentation (tray and bulk). 

Another investigation conducted by King et al. [80] found that *Salmonella* was present in processed chicken carcasses using different isolation methods, including two Australian approaches (the Australian Poultry Industry Association [APIA]: 47.8% and the Australian Standard [AS]: 37.8%) and an American method (the United States Department of Agriculture [USDA]: 10%). The researchers noted that, unlike the American method, both Australian approaches entail a priority of detecting *Salmonella,* even at low levels of contamination.

### 3.2. Victoria

According to Table A2, low levels of *S.* Typhimurium and *S.* Infantis were detected in examined eggs sourced from three farms with a previous history of *Salmonella* infection [81]. The researchers also noted that environmental contaminations of farms, as well as the physiology of the birds, such as lower body weights and higher egg production, were significant factors affecting the *Salmonella* contamination of eggs.

Milk has also been reported as a source of *Salmonella* contamination. It is usually difficult to find the source of raw milk contamination with pathogenic bacteria; however, environmental sources may play significant roles in such contaminations. In VIC, McAuley et al. [82] found that *Salmonella* was present in 7% of 15 raw milk samples from seven dairy farms (three bovine, three caprine and one ovine) located throughout the state. The sole positive sample from a bovine farm in this investigation may have been associated with a contaminated farm environment, which provided ideal conditions for *Salmonella* survival.

### 3.3. Western Australia

Rangeland goats are a common breed for meat production in the Australian goat-meat industry, exporting 90% of the products [83]. Al-Habsi et al [84] reported that 26.5% of the intestinal contents of 400 rangeland goats sampled at slaughter from four locations in WA contained *Salmonella*. *S.* Typhimurium was the most frequently isolated serovar, followed by *S.* Chester and *S.* Saintpaul, as shown in Table A2. The authors reported that high rates of *Salmonella* isolation were most likely associated with the geographic location, which can affect the duration of fasting related to ruminants’ digestive tracts’ sensitivity as well as the extent of dietary change.

Another recent investigation in this state identified *Salmonella* in 11.5% of 200 pooled eggs sourced from supermarkets and retailers in Perth city [85]. Poultry-associated serovars, including typhimurium and infantis, were recognised in this study, which indicted a noteworthy prevalence of *Salmonella* in retail egg samples in WA that might be related to a considerable number of human salmonellosis cases in recent years, relative to other states.

### 3.4. South Australia

Several investigations were conducted in SA on the occurrence of *Salmonella* in cage-laid eggs. Chousalkar et al. [86] reported that none of the 500 tested eggs sampled from caged layer farms were positive for *Salmonella*, indicating that *Salmonella* outbreaks were unlikely to be linked to the unwashed eggs collected from caged layer farms. In the next few years, another study isolated *S.* Infantis from 600 pooled cage-laid egg samples [87]. The same serovar was also identified in 3.87% of 310 pooled cage-laid egg samples in another investigation in Adelaide [88]. Although the prevalence of *Salmonella* was low in this study, the proper handling of eggs in the kitchen was suggested by the authors to reduce the probability of cross-contamination of other food materials.

In a longitudinal study of two commercial cage-layer farms by Gole et al. [89], egg samples were collected in addition to environmental samples. Over a period of 40 weeks, 10 longitudinal samplings were conducted by the researchers. *Salmonella* was present in 4% of the 521 sampled eggs and *S.* Oranienburg was reported as the predominate serovar. In another study, the prevalence of *Salmonella* during three longitudinal samplings of a layer farm with chickens at the ages of 18, 24 and 30 weeks was investigated using a different sampling strategy. The presence of *S.* Mbandaka was reported in 2.7% of 72 caged-egg samples collected at 30 weeks of lay [90]. The authors proposed regular monitoring and intervention strategies to diminish the environmental load of *Salmonella* in layer flocks to reduce the chances of eggshell contamination.

Free-range eggs have also tested positive for *Salmonella* in previous studies in SA. Moyle et al. [91] reported that 1% of 100 pooled crush egg samples obtained from two free-range flocks from two different farms contained *S.* Mbandaka. An approximately similar result (1.34%) was found for the free-range eggs tested in the study of Gole et al. [92]. Retail table eggs were also tested for the presence of *Salmonella*; 199 pooled retail egg samples (caged, free-range and barn-laid eggs) were analysed between January and June 2008, with *Salmonella* being present in 3.5% of them [93]. Infantis, Typhimurium and Johannesburg were the three identified serovars in positive samples. The authors noted the risks for consumers associated with cross-contamination and poor hygienic practices surrounding eggs during food preparation and suggested the need for the education of egg consumers about the risks.

In addition to eggs, chicken meat was also reported as positive for *Salmonella* in previous investigations in SA. As shown in Table A2, in addition to the study of Summer et al. [94], Fearnley et al. [93] also found that *Salmonella* was present in chicken meat samples sourced from supermarkets and butchers. The isolation rates were identified as higher in the skin-on (40.1%) than the skin-off samples (37.7%). *S.* Infantis was the most frequently identified serovar, accounting for 20.3% of the isolates, followed by *S.* Typhimurium phage type 135 and *S.* II Sofia. The authors cautioned that finding *S.* Infantis and *S.* Typhimurium phage type 135 in humans over the same period was a matter for concern. They recommended further investigations into causal associations for *S.* Infantis, as well as the long-term, systematic surveillance of retail foods to assess the impact of chicken meat and eggs on human salmonellosis.

As shown in Table A2, chicken portions and carcasses sampled from butcher shops, supermarkets and specialty stores in urban areas in SA were also found positive for *Salmonella*, with positive rates of 40% and 31% for the winter and summer samples respectively [79]. Further, *S.* Sofia was reported as the most prevalent serovar, accounting for 21.9% of the isolates, followed by *S.* Infantis at 8.7% and *S.* Kiambu at 3.2%. The prevalence of *Salmonella* also varied according to retail mode with rates ranging from 25% to 41.3% for chicken pieces sourced from butchers and supermarkets, respectively. The authors also mentioned that no significant differences corresponding to product type (skin-on or skin-off) and presentation (tray or bulk) were observed.

*Salmonella* was also identified in the carcasses and minced meat of kangaroos obtained from all kangaroo processing plants in SA in December 2002 and March 2004 [95]. No *Salmonella* was found in the tested samples of 2002, while around 1% of the sampled carcasses in 2004 were reported as positive for this pathogen, a finding comparable to the results (0.84%) reported by Eglezos et al. [96] in QLD. In 2002, the presence of *Salmonella* was 18% in 50 minced kangaroo meat samples. *S.* Muenchen was the common serovar identified in both kinds of samples. Among the abdominal cavities of kangaroo carcasses, 12% of the 120 tested samples contained *Salmonella* [95]. A lower frequency of *Salmonella* contamination of the outer surfaces of the carcasses than the body cavities implied that the outer surfaces were not necessarily contaminated during processing. The authors noted a minimal risk of salmonellosis associated with the consumption of kangaroo meat.

### 3.5. Queensland

Although goat meat is not commonly consumed in Australia, it is exported to Asia and the USA [97]. As shown in Table A2, a cross-sectional study of goat carcasses from two different abattoirs in Brisbane found that *Salmonella* was present in 28.9% of the samples, with *S.* Saintpaul accounting for 25% of the isolates [97]. This result shows that goat meat contaminated with faeces could be a source of human salmonellosis. The prevalence of *Salmonella* in faeces is recognised as an important risk factor for carcass contamination. Processing factors also play a significant role in carcass contamination.

In another study, Fegan et al. [98] analysed 100 pre-chill and 100 post-chill cattle carcasses from 25 consecutively slaughtered cattle belonging to unrelated groups and slaughtered at a single abattoir between March and April 2003 in QLD, which reported the presence of *Salmonella* in 2% and 3% of pre- and post-chill carcasses, respectively. Four different *Salmonella* serovars were identified in this study, of which only *S.* Muenchen was detected in both pre- and post-chill carcasses. The authors mentioned that the infrequent contamination of carcasses at this abattoir could result from the effective slaughter process and chilling practices that decreased the level of contamination.

Feral pig meat has become an alternative to the consumption of wild boar meat in some countries because of its organoleptic profiles, such as its strong flavour and dark colour [99]. As described in Table A2, feral pig carcasses obtained from a Queensland wild game processing plant between June 2004 and June 2006 were found to contain *Salmonella* using a polymerase chain reaction (PCR) [100]. This study illustrated that the low level of *Salmonella* contamination in the feral pig carcasses could arise from the implementation of hazard analysis and critical control point (HACCP)-based national quality assurance programmes, as defined by the Codex Alimentarius Commission [101].

In another investigation, Eglezos et al. [102] detected the presence of *Salmonella* in 8.7% of 300 batches of raw, frozen chicken nuggets manufactured at a chicken-processing facility in QLD between January 2003 and December 2006. *Salmonella* subspecies II (Sofia), a well-recognised serovar in the Australian poultry industry, accounted for 57.7% of the isolates. The authors indicated that raw, frozen poultry products have potential health risks for consumers if not cooked and heated appropriately.

Kangaroo meat has several nutritional properties, including low fat and high levels of conjugated linoleic acid, and can be an attractive replacement for other meat products. In a study conducted by Eglezos et al. [96], *Salmonella* was detected in less than one percent of kangaroo carcass samples obtained at two QLD processing plants between February 2003 and February 2006. The authors also found a significant relationship between the prevalence of *Salmonella* and the summer months. 

In a study of eggs and egg products (whole egg, egg pulp, egg yolk and individual farm egg pulp), *S.* Typhimurium and *S.* Infantis are among the most commonly detected *Salmonella* serovars [103]

### 3.6. Multi-States

As shown in Table A2, in a national survey of the raw meats ground beef and diced lamb from retail outlets in Sydney, Melbourne and Brisbane, *Salmonella* was present in 1.1% and 0.6% of the samples, respectively [104]. The prevalent *Salmonella* serovars were *S.* Infantis and *S.* Typhimurium, which are associated more with chicken than red meat. The authors suggested that this phenomenon may reflect cross-contamination during processing at retail points, such as butcher shops and supermarkets.

Three national surveys of the microbiological quality of beef carcasses and boneless beef have been conducted in five mainland Australian states in the past two decades. [105,106,107]. The survey conducted during the period from June to November 1998 reported the presence of *Salmonella* in both carcasses and boneless beef samples [105]. The authors noted that the infrequent findings of *Salmonella* in the beef samples in this study were attributable to the implementation and development of HACCP-based quality assurance systems in which the Australian abattoir industry had invested.

The next national survey was conducted by collecting samples from abattoirs in each of the five mainland Australian states in summer and winter, 2004. This survey was conducted to assess any changes in the microbiological quality of the tested samples since the previous survey in 1998. The results indicate that frozen boneless beef samples were positive for *Salmonella,* while no *Salmonella* was detected in the chilled beef carcasses tested [106]. The contamination rates were lower than in the previous survey, particularly for beef carcasses, because of the implementation of the HACCP systems. The authors also noted the positive effects of the implemented co-regulatory approach between industry and regulators in the Australian domestic as well as export sector.

The next national baseline microbiological survey of Australian beef was conducted in the summer and winter of 2011 on frozen boneless beef and beef primal cuts [107]. *Salmonella* was not isolated from any of the examined samples. While there were differences in sampling between this and the previous survey, there was a small but considerable improvement in the reduction in *Salmonella* in boneless meat.

According to Table A2, similar national surveys were conducted to assess the microbiological quality of Australian sheep meat. From June to November 1998, an analysis of sheep carcasses and frozen boneless sheep cuts, sourced from 15 large and medium-sized slaughterhouses and 15 boning establishments located across all states, identified the presence of *Salmonella* in both of the tested samples [108].

In the next national baseline survey, the same samples were collected from 20 abattoirs and ten boning (fabricating) establishments, accounting for approximately 78% of Australian sheep meat production. In contrast to the previous survey, lower rates of *Salmonella* recovery were observed in both carcasses (0%) and boneless products (0.5%) [109]. A minimal human health risk associated with sheep meat was reported in this investigation. The authors noted that reductions in prevalence from previous surveys were indicative of an improvement in the quality assurance environment.

The next national baseline microbiological survey of Australian sheep meat was conducted in 2011 [110]. As shown in Table A2, leg, shoulder and frozen boneless product samples sourced from 12 boning (fabricating) establishments in all Australian states were analysed. *Salmonella* was found in 2.7% of the leg samples, 0.8% of the shoulder samples and 3% of the boneless product cuts [108]. Boneless sheep meat also showed a higher *Salmonella* recovery rate (3.1%) than found in the previous survey (0.5%). The authors mentioned that the higher prevalence of *Salmonella* in the sheep trim and legs, as opposed to the carcasses, was most likely explained by the potential for cross-contamination during processing.

*Salmonella* was also present in pre-chill sheep carcasses analysed during slaughter at two Australian abattoirs, from November 2006 to March 2007 [111]. The authors noted the effectiveness of the slaughter processes at the abattoirs to minimise the contamination of carcasses, which lead to a very low public health risk of human salmonellosis.

According to recent investigations, lymph nodes are recognised as a potential reservoir of *Salmonella,* not only because of their active role in the containment of pathogens in animals, but also as a result of their unavoidable presence on carcasses during the trimming process at abattoirs [112]. A recent study of 1464 lymph nodes randomly selected from chilled cattle carcasses in five processing facilities in different Australian states (QLD, NSW, VIC, TAS and SA) found a low carriage of *Salmonella* (0.48%) amongst tested samples, with *S.* Typhimurium and *S.* Virchow being the predominant serovars [112]. *Salmonella* spp. was detected in four different anatomical sites (superficial cervical, presternal, subiliac and prepectoral), of which superficial cervical lymph nodes had the highest rate of *Salmonella* recovery (1.65%). The author suggested that the role of lymph nodes in the presence of *Salmonella* in Australian ground beef is not significant.

The incidence of *Salmonella* serovars on whole chicken carcasses before and after processing was determined by sampling from three Australian poultry abattoirs between June and December 2007 [113]. The most frequently isolated serovar was *S.* Sofia, accounting for 51% and 74% of the isolates before and after processing, respectively. The reported prevalence rates for *S.* Typhimurium, as the second most frequently isolated serovar, were 25% and 23% pre- and post-processing, respectively. Additionally, *S.* Chester, as the third isolated serovar, was only identified before processing, with a prevalence rate of 7.9%. The authors noted that the reason for higher prevalence of *S.* Sofia after processing was unclear; however, it could be related to the ability of this serovar to respond to environmental stressors and attach to surfaces.

*Salmonella* also recovered from 26.5% of 200 pooled caecal samples of Australian meat chickens obtained from 20 poultry abattoir plants owned by seven commercial companies throughout the country. *S.* Sofia (34%) was reported as the most frequently isolated serovar among 12 different identified serovars in this investigation [114].

It has been estimated that approximately 730 million servings of sausages are consumed by Australians annually and there is an increase in the use of pork minced meat resulting from consumer preferences. Although pork-related foodborne outbreaks from the food service sector in Australia are mostly caused by *Salmonella* [115], there has been limited information over the past few decades pertaining to the prevalence of *Salmonella* in pork products. In a study by Hamilton et al. [116], fresh pork sausages and fresh pork mince samples sourced from butcher shops and supermarkets located in the five largest capital cities in Australia were found positive for *Salmonella,* as shown in Table A2.

## 4. Antimicrobial Resistance Patterns of Non-Typhoidal *Salmonella* at the Human–Food Interface in Australia

Antimicrobial resistance in non-typhoid *Salmonella* is an important problem worldwide, resulting from the indiscriminate use of antibiotics in both animal and human populations [117]. According to surveillance data, there has been an obvious increase in the overall resistance of different *Salmonella* serovars to different antimicrobials over the past few decades [118,119]. Typically, salmonellosis does not need any antibiotic treatment; however, in some severe systemic salmonellosis, antibiotics may be administered [118]. Fluoroquinolones and extended spectrum cephalosporins are used as the drugs of choice for the treatment of human salmonellosis [120]. However, the rise in antimicrobial-resistant *Salmonella,* especially to antimicrobials commonly prescribed to treat human salmonellosis, has become a significant global public health concern [121,122,123].

It is difficult to compare the prevalence of antimicrobial resistance in different parts of the world for several reasons, including the heterogeneity of sampling sources, testing methods and the interpretation of results [114]. However, the overall prevalence of antimicrobial resistance in Australia is considerably lower than in other parts of the world, particularly in terms of critically important antimicrobials for the treatment of human salmonellosis [121,122,123], which could be associated with the conservative approach of applying antibiotics to food-producing animals in Australia [124,125]. In this manuscript, we review the resistance patterns of non-typhoidal *Salmonella* in humans, foods of animal origin and food-producing animals in Australia with a focus on critically important antimicrobials that are commonly used for the treatment of non-typhoidal *Salmonella* infections in humans. A summary of the antimicrobial resistance trends can be found in Table A3.

### 4.1. New South Wales

In NSW, nalidixic acid and amikacin were found to be effective for all *Salmonella* isolates recovered from diarrhoeal calves using the Kirby–Bauer disc diffusion method [126]. However, 25% of the isolates were resistant to streptomycin and 21.1% to combination sulphonamides, while resistance to tetracycline and sulfamethoxazole/trimethoprim was 11.8%. Moreover, a low rate of resistance (1–18%) has also been reported against ampicillin, neomycin, kanamycin and apramycin as given in Table A3. Multidrug resistance was also identified in 14.3% of the isolates. In the study, the majority of isolates (72.4%) were susceptible to commonly used veterinary antibiotics, yet resistance to a critically important antibiotic, ceftiofur (1.3%), was also observed. Moreover, the finding of multi-drug-resistant isolates in dairy beef operations was also a matter for concern.

The isolates recovered from food animals between 2007 and 2011 in NSW were screened for susceptibility to 18 antimicrobials by the disc diffusion method, using the calibration dichotomous susceptibility test [127]. A total of 66.1% of isolates were susceptible to all antimicrobials, while 8.5% of the isolates were resistant to four or more antibiotics. As shown in Table A3, the highest resistance rate was found against sulfafurazole, followed by ampicillin, tetracycline and trimethoprim. No resistance was reported to fluoroquinolones or third generation cephalosporins, as commonly used antimicrobials for the treatment of non-typhoidal human salmonellosis. This could be because of the conservative approach of applying antibiotics to food-producing animals in Australia. The use of first-line antibiotics, such as fluoroquinolones, for human salmonellosis is banned and ceftiofur is not approved for mass administration in food-producing animals [124,125]. The authors declared a low level of antimicrobial resistance with no resistance to critical antimicrobials for human treatment among *Salmonella* isolates recovered from clinically ill food animals in NSW.

### 4.2. Victoria

Reports by McAuley et al. [128] in VIC indicated that no antimicrobial resistance was identified among the *Salmonella* isolates from dairy farm environments, in agreement with another Australian study on the faeces of dairy cattle at abattoirs [129].

### 4.3. Western Australia

In this state, Al-Habsi et al. [84] reported that *Salmonella* isolates from rangeland goats’ faecal samples remained susceptible to the critically important antimicrobials tested, including ceftiofur, ceftriaxone, chloramphenicol and ciprofloxacin.

Non-susceptibility was most frequently identified for azithromycin, followed by other antibiotics (Table A3). 

In another study in WA on retail table eggs, only two (6.7%) of *S.* Typhimurium isolates were found to be resistant to ampicillin, representing minimal public health risks associated with antimicrobial-resistant *Salmonella* isolates from retail table eggs [85]. This study’s finding regarding the absence of resistance to critically important antimicrobials for the treatment of human salmonellosis was observed to be similar to the findings of previous Australian investigations.

### 4.4. South Australia

In SA, *Salmonella* subsp. 2 ser 21: z10: z6 (Wandsbek) showed non-susceptibility to ampicillin and cephalothin, while intermediate resistance to florphenicol was reported for this serovar. In contrast, although all antimicrobials were found to be effective for *S.* Bovismorbificans isolates, intermediate resistance to ampicillin was identified for *S.* Agona isolates [130]. The authors noted that the serovar might play a significant role in determining antibiotic resistance, which requires further investigation.

### 4.5. Multi-States

*Salmonella* spp. isolated from commercial caged layer flocks in NSW and SA showed high susceptibility (91.72%) to all antimicrobials tested. Limited non-susceptibility was identified for the antibiotics shown in Table A3 [131]. Antimicrobials including cefotaxime, ceftiofur, ciprofloxacin, chloramphenicol, gentamycin, neomycin and streptomycin were reported to be effective for all *Salmonella* isolates from caged layer flocks [131]. Similar to the study by Abraham et al. [127] in NSW, no resistance was observed to fluoroquinolones or extended spectrum cephalosporins, which contrasted with global reports for these antimicrobials in *Salmonella* isolated from food animals [132]. 

In another study, *Salmonella* isolates from cattle populations at slaughter (dairy and beef cattle and veal calves) collected from 31 abattoirs, representing 85% of total beef exports, indicated resistance to only florfenicol in 34.7% and 38.9% of the isolates from dairy cattle and veal calves, respectively, while there was no resistance to fluoroquinolones or cephalosporins. Conversely, according to Table A3, the beef cattle isolates not only showed resistance to florfenicol but also to tetracycline, streptomycin and ampicillin, as well as to trimethoprim/sulfamethoxazole [129]. The finding of no resistance to cephalosporins and fluoroquinolones in this study reinforced the results of other similar Australian investigations [127,131]. Multidrug-resistant traits were found only in a small portion (7.5%) of beef cattle isolates. The authors mentioned that the low level of resistance in *Salmonella* isolates to important antimicrobials in human medicine represented a minimal public health risk.

In a pilot national survey throughout Australia between July and December 2015, Kidsley et al. [133] discovered that the isolates from caecal contents of healthy slaughter-age pigs showed resistance to streptomycin, tetracycline, ampicillin, trimethoprim/sulfamethoxazole and chloramphenicol with resistances ranging from 7.3% to 22%. Lower rates of non-susceptibility also found for amoxicillin/clavulanate and gentamicin. The authors reported that non-susceptibility to first-line antimicrobials is common among *Salmonella* spp. isolates from healthy slaughter-age pigs in Australia; however, as in previous studies, no resistance in *Salmonella* isolates to antimicrobials critically important for human infections was observed.

As a part of an international study conducted by Aarestrup et al. [134], the antimicrobial susceptibility of 46 *S.* Weltevreden isolates originating from humans, animals, food products and the environment was investigated. The results indicate that 2.2% of the isolates were resistant to the ampicillin, streptomycin and sulfamethoxazole, as well as to tetracycline and trimethoprim. Nonetheless, no resistant isolate was reported for nalidixic acid, neomycin, chloramphenicol and florfenicol. Such relatively low resistance rates may result from the restricted use of antimicrobials in Australia.

Fegan et al. [135] discovered that the majority of *Salmonella* isolates (76%) from beef cattle presented for slaughter at abattoirs across Australia between September 2002 and January 2003 were sensitive to all antimicrobials tested. However, a few *S.* Typhimurium isolates recovered from lot-fed cattle showed resistance to ticarcillin/clavulanic acid, trimethoprim/sulfamethoxazole and trimethoprim, as well as to amikacin, ampicillin, ceftazidime and nitrofurantoin. In contrast, *S.* Give from grass-fed cattle was reported to be resistant to cephalothin. The authors did not suggest a conclusion concerning the effects of the production system on antibiotic resistance as indicated by the low number of resistant isolates.

Recent reports from Abraham et al. [114] indicated that all *Salmonella* isolates from pooled caecal samples of chickens at slaughter collected from 20 poultry abattoirs of seven commercial companies in Australia were susceptible to ceftiofur, chloramphenicol, ciprofloxacin, colistin, florfenicol, gentamicin and tetracycline, whereas only *S.* Sofia isolates exhibited resistance rates of 1.9%, 3.8% and 11.3% to streptomycin, ampicillin and cefoxitin, respectively. No multidrug-resistant isolate was found in this study. Similar to previous Australian studies [120,124], this study also emphasised the absence of *Salmonella* isolates resistant to critically important antimicrobial agents.

## 5. Food-Related Origin of *Salmonella* Infections/Outbreaks in Australian States

Generally, eggs and egg-based products were the most commonly reported food vehicle in Australian foodborne *Salmonella* outbreaks [136,137]. Fifty percent of *Salmonella* outbreaks in Australia have been attributed to egg and egg-based foods such as Vietnamese style rolls, sandwiches, and salads. From 2001 to 2016, egg-based sauces, desserts, sandwich and salads containing raw or lightly cooked eggs were frequently reported to be associated with *Salmonella* outbreaks; however, the frequency of reported outbreaks related to the other animal-source foods including poultry meat, beef and pork were declined over the time period [138]. Nevertheless, in a recent study, chicken meat is claimed to be associated with 43.7% of the *Salmonella* cases in QLD [139].

## 6. Management Strategies

Foodborne human salmonellosis reports have increased over the past two decades in Australia, which has one of the highest rates compared to similar developed countries [140]. There were an estimated 56,200 cases of salmonellosis, with 72% of these considered to be foodborne between 1991 and 2014 [140]. A One Health approach including multiple interventions is necessary to better understand, prevent, and control of *Salmonella* and its infections, which shows that the health of people is completely related to the health of animals and the environment. Applying biosecurity interventions at farm level particularly poultry farms to protect the primary production level against the introduction of *Salmonella* should be effective based on a good understanding of the regional risk factors at the farm level. 

Since 2013, a wide range of regional risk management activities have been implemented by state and territory health, agriculture and food authorities, especially in the poultry and egg industry [140]. In addition, some states are developing their own foodborne illness reduction strategies. For instance, Food Authorities in NSW and WA have developed a plan to reduce foodborne illnesses including salmonellosis by 30% by 2021 [49,141]. 

## 7. Conclusions

Non-typhoidal *Salmonella,* as an important zoonotic pathogen, not only causes disease and death but also leads to several socio-economic losses. Non-typhoidal *Salmonella* infections are particularly associated with vulnerable groups of people, such as the elderly and children, and is mostly recovered from animal food sources, especially eggs and egg-based products. The majority of food-related salmonellosis outbreaks in Australia occur in crowded public places such as restaurants and cafés, events and functions, as well as child-care and aged-care centres. Therefore, strict food-safety principles are required to prevent and control non-typhoidal *Salmonella* infections at events and public gathering along with restaurants and cafés. Such measures depend on the close collaboration of different food-related sectors from farm to fork.

This review also highlights the antimicrobial resistance patterns of non-typhoidal *Salmonella* isolated at the human–food–environment interface with a focus on clinically important antimicrobials in human treatment. Although no considerable alarming trend related to such antimicrobials has been reported in this country, a low level of resistance to less critical antimicrobials, such as cefoxitin, trimethoprim/sulfamethoxazole, ampicillin and tetracycline, has been reported in different Australian states. Nevertheless, proactive monitoring of antimicrobial resistance is highly recommended by Australian researchers to ensure the long-term protection of both human and animal health.

According to the evidence gathered in this review, we believe that for the control and prevention of salmonellosis outbreaks in Australia, in addition to improving routine monitoring and surveillance and the application of effective intervention strategies at the farm level, strict food-safety management is required to reduce the level of cross-contamination in processing sectors and kitchens.

## Appendix A

**Table A1:** Human salmonellosis outbreaks caused by suspected food of animal origin in Australia.

State/Region	Year	Place	Age Range	No. of Cases with Symptoms	No. of Lab-Confirmed Cases	No. of Hospitalisations	No. of Deaths	Suspected Source(s)	Serotype(s)	Reference
NSW	2018–19	Metropolitan Sydney region	-	193	-	-	-	13 NSW poultry egg facilities	Enteritidis	[49]
	2015	Shopping complex	3–45	19	6	1	-	Cooked-tuna sushi rolls	Agona	[16]
	2015	Bakery	1–77	26	19	-	-	Vietnamese bread rolls containing pork or chicken with chicken-liver pâté and raw egg mayonnaise filling	Typhimurium	[30]
	2014	High-profile sports club	-	35	10	-	-	Lamb meal supplied by an external caterer	Typhimurium	[31]
	2011	Vietnamese bakery	1–75	83	47	20	-	Chicken, pork and salad rolls	Typhimurium	[29]
	2010	Kebab takeaway shop	7–70	45	31	8	-	Chicken kebab roll containing hummus and tabbouleh	Typhimurium	[47]
	2009	Privately catered barbeque at a sport club	1–70	71	30	13	-	Raw egg mayonnaise used in a Russian salad	Typhimurium	[32]
	2008	Private seller/restaurant	3–91	-	44	6	-	Egg/egg-containing dishes	Typhimurium	[33]
	2008	Aged-care facility	median age 81.5 years	10	8	-	-	Raw egg dessert	Typhimurium	[26]
	2007	Bakery	1–74	365	319	136	-	Mayonnaise-containing raw egg	Typhimurium	[27]
	2005	-	11–81	-	37	-	-	Lambs’ liver	Typhimurium	[36]
	2002	Restaurant	1–77	17	12	2	-	Shell egg-based salad dressings	Potsdam	[34]
VIC	2002	Bakery	-	-	10	-	-	Contaminated eggs or cream	Typhimurium	[50]
	2001	Hotel buffet	7–72	18	4	2	-	Lambs’ fry	Typhimurium	[14]
SA	2017–18	-	1–91	-	25	10	-	Free-range eggs	Hessarek	[51]
	2009	Wedding	2–90	30	9	-	-	Garlic aioli containing raw egg yolk	Typhimurium	[58]
	2005	Catered luncheons	21–63	61	32	-	-	Bread rolls contaminated with chicken	Typhimurium	[57]
	2003	Commercial food outlets	3–82	-	6	-	-	Cheesecake containing eggs	Typhimurium	[56]
	2001	Aged-care facility	-	18	13	3	-	Pie glaze containing raw egg	Typhimurium	[54]
	2001	Christmas function	-	-	11	4	-	Eggs used in preparing tiramisu	Typhimurium	[53]
	2001	Italian restaurant	26–31	-	2	-	-	Unrecognised	Zanzibar	[55]
	2000	Chinese restaurant	1–77	-	6	-	-	Unrecognised	Typhimurium	[142]
QLD	2007	Restaurants	1–54	-	44	-	-	Egg	Typhimurium	[143]
	2003	Restaurant	2–75	19	13	-	-	Roast pork	Typhimurium	[60]
	2004	Brisbane CBD (lunch meal)	20–39	13	11	-	-	Sushi	Singapore	[61]
	2002	Child-care centre	1–5	16	10	-	-	Egg sandwich	Typhimurium	[59]
WA	2000	Religious festival celebration	1–73	53	14	-	-	Mock ice-cream dessert containing raw eggs	Typhimurium	[144]
TAS	2007	Bakery	NA	-	18	-	-	Bakery products	Typhimurium	[63]
		Retail businesses	NA	-	2	-	-	Eggs or dishes containing eggs	Typhimurium	
	2008	Restaurant	NA	-	47	-	-	Chicken sandwiches containing aioli	Typhimurium	
	2005	Group functions and restaurants	1–86	-	125	-	-	Products containing raw egg	Typhimurium	[62]
ACT	2012	Café	19–62	-	20	2	-	Eggs benedict	Typhimurium	[64]
	2009	Restaurant	9–71	20	9	-	-	Tiramisu dessert containing raw egg	Typhimurium	[66]
	2008	Restaurant	3–53	24	16	2	-	Eggs and hollandaise sauce	Typhimurium	[65]
NT	2017	Aboriginal community	3–63	22	7	2	-	Sea-turtle meat	Muenchen	[17]
	2015	Restaurant	16–74	21	9	7	-	Duck prosciutto	Typhimurium	[15]
	2009	Car rally	21–72	76	-	-	-	Barramundi fillets	Litchfield	[69]

**Table A2:** Occurrence of non-typhoidal *Salmonella* in foods of animal origin.

State	Product	Sample size	Percentage Positive	Predominant Serotype(s)	Detection Method	Reference
NSW	Chicken meat	549	47.70	Sofia Infantis Typhimurium Kiambu Subsp. 1	Cultural	[79]
	Chicken carcasses	90	47.80	*Salmonella* spp.	APIA	[80]
		90	37.80		AS	
		90	20.00		USDA	
VIC	Eggs	8958	0.002	Typhimurium		[81]
			0.005	Infantis		
	Raw milk	15	7.00	*Salmonella* spp.	Cultural	[82]
SA	Free-range eggs	-	1.34	*Salmonella* spp.	Cultural	[92]
	Pooled shell crush	100	1.00	Mbandaka	Cultural	[91]
	Caged eggs	72	2.70	Mbandaka	Cultural	[90]
	Cage-laid eggs	521	4.00	Oranienburg Worthington Typhimurium	Cultural—quantitative polymerase chain reaction (PCR)	[89]
	Cage-laid eggs	310 pooled samples	4.50	Infantis	Cultural	[88]
	Cage-laid eggs	60 pooled sample	7.00	Infantis	Cultural	[87]
	Retail chicken meat	365	38.80	Infantis Typhimurium Sofia Kiambu	Cultural, ELISA kit	[93]
	Retail table eggs	199 pooled samples	3.50	Infantis Typhimurium Johannesburg		
	Cage-laid eggs	500	0.00	-	Cultural	[86]
	Whole chicken, skinless breasts, livers	260	53.70	-	Cultural	[92]
	Kangaroo carcasses	60	0.00	-	Cultural	[95]
		385	1.04	Muenchen Singapore		
	Minced kangaroo meat	50	18.00	Muenchen Havana		
	Abdominal cavities of kangaroo carcasses	120	12.00	Singapore Zehlendorf Infantis Fremantle Anatum Sofia Kottbus Rubislaw		
QLD	Chicken meat	310	35.50	Sofia Infantis Typhimurium Kiambu Subsp. 1	Cultural	[79]
	Goat carcasses	121	28.90	Saintpaul Typhimurium Chester Agona	Cultural	[97]
	Feral pig carcasses	217	1.38	*Salmonella* spp.	Automated PCR	[100]
	Frozen chicken nuggets	300 batch	8.70	*Salmonella* subsp. I *Salmonella* subsp. II (Sofia) Typhimurium	Cultural	[102]
	Kangaroo carcasses	836	0.84	Emmastad Rubislaw Eastbourne Muenchen Havana Saintpaul Reading	Automated PCR	[96]
	Pre-chill cattle carcasses	100	2.00	Muenchen	Cultural	[98]
	Chilled cattle carcasses	100	3.00	Bredeney Give Mbandaka Muenchen		
	Egg and egg products (whole egg, egg pulp, egg yolk and individual farm egg pulp)	1031	32.00	Singapore Mbandaka Cerro Typhimurium Infantis	Cultural	[103]
WA	Pooled caecal samples	200	26.50	Sofia Abortusovis Adelaide Typhimurium		[114]
	Retail table eggs	200 pooled samples	11.50	Typhimurium Infantis	Cultural	[85]
	Intestinal contents of rangeland goats	400	26.50	Typhimurium Chester Saintpaul		[84]
Multi-States	Lymph nodes of beef cattle carcasses	1464	0.48	Typhimurium Virchow Dublin Kentucky Chailey	BAX PCR assay	[112]
	Sheep and lamb legs	613	2.70	Bovismorbificans Adelaide Saintpaul Typhimurium Havana Newport Tennessee Chester Kottbus Infantis Hvittingvoss	Cultural	[110]
	Shoulders	613	0.80			
	Frozen boneless sheep/lamb products	551	3.00			
	Frozen boneless beef	1165	0.00	*Salmonella* spp.	Cultural	[107]
	Beef primal cuts	1144	0.00			
	Fresh pork sausages	116	8.60	*Salmonella* spp.	Cultural	[116]
	Fresh pork mince	148	1.50			
	Pre-chill sheep carcasses	164	1.30	*Salmonella* spp.	Automated immunomagnetic separation	[111]
	Chicken carcasses	90	99.00	Sofia Typhimurium Chester	Cultural	[113]
		180	38.30			
	Ground beef	360	1.10	Typhimurium	Cultural	[104]
	Diced lamb	360	0.60	Infantis Typhimurium		
	Chilled sheep carcasses	1117	0.00	*Salmonella* spp.	Cultural	[109]
	Frozen boneless sheep meat	560	0.50			
	Chilled beef carcasses	1155	0.00	*Salmonella* spp.	Cultural	[106]
	Frozen boneless beef	1082	0.09			
	Beef carcasses	1275	0.20	*Salmonella* spp.	Cultural	[105]
	Frozen boneless beef	990	0.10			
	Sheep carcasses	917	0.10	*Salmonella* spp.	Cultural	[108]
	Frozen boneless sheep meat	467	1.30			

**Table A3:** Antimicrobial resistance of non-typhoidal *Salmonella* isolated at the human–food interface of Australia.

State/ Region	Source	Species/Serotype	Antibiotic	Resistance %	Applied Method	Reference
NSW	Diarrhoeal calves’ faeces	*Salmonella* spp.	Ampicillin	18.4	Kirby–Bauerdisc diffusion	[126]
			Combination sulphonamides	21.1		
			Tetracycline	11.8		
			Sulfamethoxazole/trimethoprim	11.8		
			Neomycin	13.2		
			Ceftiofur	1.3		
			Kanamycin	9.2		
			Apramycin	5.3		
			Amoxycillin/clavulanic acid	1.3		
			Streptomycin	25		
			Nalidixic acid	0		
			Amikacin	0		
	Livestock	*Salmonella* spp.	Amoxicillin/clavulanic	0		[127]
			Cefalexin	0		
			Cefoxitin	0		
			Cefotaxime	0		
			Cefepime	0		
			Nalidixic acid	0		
			Ciprofloxacin	0		
			Imipenem	0		
			Azithromycin	0		
			Sulfafurazole	28.5		
			Ampicillin	17		
			Tetracycline	15.8		
			Trimethoprim	8.5		
			Neomycin	4.2		
			Apramycin	3		
			Chloramphenicol	2.4		
			Gentamicin	1.2		
			Ticarcillin/clavulanic acid	0.6		
VIC	Bovine, ovine and caprine dairy farm environments	Orion Infantis Zanzibar	Amoxicillin and clavulanic acid		NA	[128]
			Ampicillin	0		
			Cefazolin	0		
			Cefotaxime	0		
			Cefoxitin	0		
			Ceftiofur	0		
			Ceftriaxone	0		
			Chloramphenicol	0		
			Ciprofloxacin	0		
			Chloramphenicol	0		
			Gentamicin	0		
			Kanamycin	0		
			Meropenem	0		
			Nalidixic acid	0		
			Streptomycin	0		
			Tetracycline	0		
			Sulfamethoxazole/trimethoprim	0		
SA	Backyard chicken’s faeces	*Salmonella* subsp. 2 ser 21: z10: z6 (Wandsbek)	Ampicillin	-	Disc diffusion	[130]
			Cephalothin	-		
WA	Retail table eggs	Typhimurium	Ampicillin	6.7	Micro-broth dilution	[85]
		Typhimurium Infantis	Sulfamethoxazole	0		
			Trimethoprim	0		
			Gentamicin	0		
			Azithromycin	0		
			Ciprofloxacin	0		
			Nalidixic Acid	0		
			Tetracycline	0		
			Tigecycline	0		
			Meropenem	0		
			Ceftazidime	0		
			Cefotaxime	0		
			Colistin	0		
			Chloramphenicol	0		
	Intestinal content of rangeland goats	*Salmonella* spp.	Azithromycin	14.2	Broth microdilution assay	[84]
			Tetracycline	10.5		
			Ampicillin	5.7		
			Amoxicillin–clavulanate	3.8		
			Cefoxitin	3.8		
			Trimethoprim/sulfamethoxazole	1.9		
			Gentamicin	0.9		
			Streptomycin	0.9		
Multi-States	Beef cattle faeces		Florfenicol	29.2		[129]
			Streptomycin	7.5		
			Ampicillin	7.5		
			Trimethoprim/sulfamethoxazole	7.5		
			Tetracycline	6.6		
			Cephalosporins	0		
			Fluoroquinolones	0		
	Dairy cattle faeces		Florfenicol	34.7		
			Cephalosporins	0		
			Fluoroquinolones	0		
	Veal calf faeces		Florfenicol	38.9		
			Cephalosporins	0		
			Fluoroquinolones	0		
	Caecal contents of slaughter-age pigs	*Salmonella* spp.	Ampicillin	20.3	Micro-broth dilution	[133]
			Tetracycline	26.1		
			Chloramphenicol	7.3		
			Trimethoprim/sulfamethoxazole	11.6		
			Amoxicillin/clavulanate	2.9		
			Gentamicin	2.9		
			Streptomycin	22		
			Ceftiofur	0		
			Cefoxitin	0		
			Ceftriaxone	0		
			Ciprofloxacin	0		
	Caged layer flocks	Mbandaka	Amoxycillin	6.66	Broth microdilution	[131]
			Ampicillin	6.66		
			Cephalothin	3.33		
			Tetracycline	16.66		
			Trimethoprim	3.33		
		Typhimurium	Amoxycillin	3.84		
			Ampicillin	3.84		
			Tetracycline	3.84		
		Worthington	Amoxycillin	16.12		
			Ampicillin	16.12		
			Cephalothin	6.45		
	Lot-fed cattle	Typhimurium	Ticarcillin/clavulanic acid	1 isolate	VITEK Juniorsystem	[135]
			Trimethoprim/sulfamethoxazole			
			Trimethoprim			
		Typhimurium	Amikacin	1 isolate		
			Ampicillin			
			Ceftazidime			
		Typhimurium	Nitrofurantoin	1 isolate		
		Typhimurium	Ticarcillin/clavulanic acid	1 isolate		
	Grass-fed cattle	Give	Cephalothin	1 isolate		
	Human Animal food products	Weltevreden	Ampicillin	2.2	MIC	[134]
			Streptomycin	2.2		
			Sulfamethoxazole	2.2		
			Tetracycline	2.2		
			Trimethoprim	2.2		
			Nalidixic acid	0		
			Neomycin	0		
			Chloramphenicol	0		
			Florphenicol	0		
	Caecal samples of chickens at slaughter	Sofia	Streptomycin	1.9	MIC	[114]
			Ampicillin	3.8		
			Cefoxitin	11.3		
		*Salmonella* spp.	Ceftiofur	0		
			Chloramphenicol	0		
			Ciprofloxacin	0		
			Colistin	0		
			Florfenicol	0		
			Gentamicin	0		
			Tetracycline	0		
			Trimethoprim/sulf	0		
			Ceftriaxone	0		
			Amoxicillin–clavulanate	0		
